# Effect of Currently Approved Carriers and Adjuvants on the Pre-Clinical Efficacy of a Conjugate Vaccine against Oxycodone in Mice and Rats

**DOI:** 10.1371/journal.pone.0096547

**Published:** 2014-05-05

**Authors:** Marco Pravetoni, Jeffrey S. Vervacke, Mark D. Distefano, Ashli M. Tucker, Megan Laudenbach, Paul R. Pentel

**Affiliations:** 1 Minneapolis Medical Research Foundation, Minneapolis, Minnesota, United States of America; 2 University of Minnesota, School of Medicine, Department of Medicine, Minneapolis, Minnesota, United States of America; 3 University of Minnesota, School of Medicine, Department of Pharmacology, Minneapolis, Minnesota, United States of America; 4 University of Minnesota, Center for Immunology, Minneapolis, Minnesota, United States of America; 5 University of Minnesota, Department of Chemistry, Minneapolis, Minnesota, United States of America; University of Strathclyde, United Kingdom

## Abstract

Vaccination against the highly abused prescription opioid oxycodone has shown pre-clinical efficacy for blocking oxycodone effects. The current study further evaluated a candidate vaccine composed of oxycodone derivatized at the C6 position (6OXY) conjugated to the native keyhole limpet hemocyanin (nKLH) carrier protein. To provide an oxycodone vaccine formulation suitable for human studies, we studied the effect of alternative carriers and adjuvants on the generation of oxycodone-specific serum antibody and B cell responses, and the effect of immunization on oxycodone distribution and oxycodone-induced antinociception in mice and rats. 6OXY conjugated to tetanus toxoid (TT) or a GMP grade KLH dimer (dKLH) was as effective as 6OXY conjugated to the nKLH decamer in mice and rats, while the 6OXY hapten conjugated to a TT-derived peptide was not effective in preventing oxycodone-induced antinociception in mice. Immunization with 6OXY-TT s.c. absorbed on alum adjuvant provided similar protection to 6OXY-TT administered i.p. with Freund’s adjuvant in rats. The toll-like receptor 4 (TLR4) agonist monophosphoryl lipid A (MPLA) adjuvant, alone or in combination with alum, offered no advantage over alum alone for generating oxycodone-specific serum antibodies or 6OXY-specific antibody secreting B cells in mice vaccinated with 6OXY-nKLH or 6OXY-TT. The immunogenicity of oxycodone vaccines may be modulated by TLR4 signaling since responses to 6OXY-nKLH in alum were decreased in TLR4-deficient mice. These data suggest that TT, nKLH and dKLH carriers provide consistent 6OXY conjugate vaccine immunogenicity across species, strains and via different routes of administration, while adjuvant formulations may need to be tailored to individual immunogens or patient populations.

## Introduction

Drug addiction is a worldwide public health concern [1]. Abuse of prescription opioid analgesics is highly prevalent in the USA with oxycodone and hydrocodone being amongst the most commonly abused drugs in people over 12 years of age [2]. In the USA, overdose is the leading cause of death after prison release, with prescription opioids (oxycodone and hydrocodone) being the most common substances involved [3]. To address this problem, vaccination against drugs of abuse may offer a complementary treatment strategy to current addiction therapies.

Addiction vaccines are made by conjugating the target drug to a larger immunogenic carrier peptide or protein of bacterial, viral or other foreign origin and by the use of adjuvants to increase immunogenicity. Drugs of abuse are not immunogenic on their own due to their small size, and the larger carrier is thought to provide signaling for T cell-dependent B cell activation [4]. Vaccine efficacy is limited by the ability of generating high levels of high affinity drug-specific serum antibodies that reduce drug distribution to the brain and block drug-induced behavioral effects.

Vaccine development is largely based on empirical optimization of the various elements composing the final injectable formulation. Several carrier and adjuvant options need to be considered to provide good manufacturing practice (GMP) grade and cost effective vaccines or to generate individualized vaccine formulations targeting different patient populations. Recent studies highlighted the importance of evaluating hapten design, choice of carrier, adjuvant and delivery platform to enhance the immunogenicity and efficacy of vaccines against drugs of abuse [5]–[14].

In a series of conjugate vaccines showing varying degrees of pre-clinical efficacy against prescription opioids [14], [15], the lead immunogen was composed of a hapten based on derivatization of oxycodone at the C6 position (6OXY) and conjugated through covalent amide bond to the native keyhole limpet hemocyanin (nKLH) carrier protein [14]. The nKLH, a large multi-subunit decamer (MW∼5–8 million Da), is a highly immunogenic carrier that has shown clinical safety [16]. Vaccination of mice and rats with the 6OXY-nKLH in Freund’s and alum adjuvants was effective in blocking oxycodone and hydrocodone distribution to brain and behavioral effects [14].

Here, to provide clinically viable vaccine formulations of 6OXY-nKLH and to further improve its efficacy, we studied the effect of conjugating the 6OXY hapten to alternative carriers and the use of different adjuvants on generation of oxycodone-specific serum antibody titers, and their efficacy reducing oxycodone distribution to the brain and oxycodone-induced nociception in mice and rats. Additionally, we tested if analysis of B cell responses to vaccination may help to understand the mechanisms underlying vaccination efficacy and aid rational vaccine design. To this end, we adapted a novel enrichment method paired to multicolor flow cytometry [17]–[19] to detect and analyze rare hapten-specific B cells within the whole B cell repertoire [20].

In the current study, we conjugated the 6OXY hapten to the clinically approved tetanus toxoid (TT), to a TT-derived peptide previously shown to be an effective carrier for small molecule haptens [21] and to a GMP grade KLH dimer (dKLH). We then tested the immunogenicity and efficacy of these conjugate immunogens using Freund’s adjuvant or the clinically approved alum and monophosphoryl lipid A (MPLA) adjuvants in mice or rats using either the s.c. or i.p. route of administration. The MPLA adjuvant is a toll-like receptor 4 (TLR4) agonist that induces robust Th_1_ activation, but in the current study MPLA negatively affected the immunogenicity of 6OXY-containing immunogens. TLRs are expressed on antigen-presenting cells and B cells, and modulate adaptive immune responses against a variety of pathogens or xenobiotics [22]. Here, we explored the relevance of TLR4 signaling to vaccination with 6OXY-nKLH using TLR4-deficient mice. These data provide evidence, and potential underlying mechanisms, of how certain approved carriers and adjuvants mediate the pre-clinical efficacy of a vaccine against oxycodone.

## Materials and Methods

### 2.1 Drugs and reagents

Oxycodone was obtained through the NIDA Drug Supply Program and Sigma (St. Louis, MO). Oxycodone doses and concentrations are expressed as the weight of the base. Alum (Alhydrogel85, Brenntag Biosector, (Frederikssund, Denmark), Freund’s complete (Calbiochem, San Diego, CA) and incomplete adjuvant (Sigma-Aldrich, St. Louis, MO), and monophosphoryl lipid A (MPLA SM VacciGrade, InvivoGen, San Diego, CA) were used according to manufacturer instructions.

### 2.2 Synthesis of oxycodone-based 6OXY hapten

The oxycodone-based hapten with a tetraglycine linker at the C6 position of the morphinan structure (6OXY) was synthesized as previously described [13], [15].

### 2.3 6OXY hapten conjugation to carrier proteins

For vaccination studies 6OXY was conjugated to the native keyhole limpet hemocyanin (nKLH, Thermo Fisher, Rockford, IL), to the GMP dimer KLH (dKLH, Stellar Biotechnologies, Port Hueneme, CA) or to tetanus toxoid (TT, U Mass Biologics, MA) using carbodiimide coupling as described before [13], [15]. For ELISA, 6OXY was conjugated to OVA (Sigma Aldrich). For enrichment of hapten-specific B cells paired to flow cytometry analysis, 6OXY was conjugated to Red-Phycoerytrin (PE, Prozyme, Hayward, CA).

### 2.4 Synthesis of TT-derived peptide and conjugation to 6OXY hapten

6OXY was conjugated to a TT-derived peptide with the following sequence: PDAQLVPGINGKAIHLVNNESSE, which has been successfully used as a carrier for benzopyrene haptens [21].

#### 2.4.1 Synthesis of PDAQLVPGINGKAIHLVNNESSE-OH

Peptide synthesis was carried out using an automated solid-phase peptide synthesizer (PS3, Protein Technologies Inc, Memphis, TN) employing standard Fmoc/HCTU based chemistry. Synthesis began on preloaded Fmoc-Glu(OtBu)-Wang resin (0.10 mmol) and the peptide chain was elongated using HCTU/N-methylmorpholine-catalyzed, single coupling steps with 4 eq of both protected amino acids and HTCU for 30 min. Following complete chain elongation, the peptide’s N-terminus was deprotected with 10% piperidine in DMF (v/v) and the presence of the resulting free amine was confirmed by ninhydrin analysis. The resin containing the peptide was washed with CH_2_Cl_2_, dried *in vacuo* overnight, weighed, and divided into two portions for further synthesis on a reduced scale. Using 50.0 µmol of peptide, the free amino terminus was acylated with the oxycodone analog (20 mg, 50 µmol, 1eq) catalyzed by DIEA (8.6 µL, 5.0 µmol, 0.1 eq) in DMF (5 mL) for 10 h. After acylation was judged complete by ninhydrin analysis, the resin bound peptide was washed thoroughly with CH_2_Cl_2_ and dried *in vacuo* for 4 h. The peptide was cleaved from the resin along with simultaneous side chain deprotection by treatment with Reagent K containing TFA (10 mL), crystalline phenol (0.5 g), 1,2-ethanedithiol (0.25 mL), thioanisole (0.5 mL), and H_2_O (0.5 mL) for 2 h at rt. The released peptide was collected and combined with TFA washes of the resin before precipitation of the peptide in chilled Et_2_O (100 mL). The crude solid peptide was collected by centrifugation, the supernatant was removed, and the resulting pellet was washed 2 times with cold Et_2_O (50 mL) repeating the centrifugation and supernatant removal steps each time. The crude peptide was purified using a semipreparative C_18_ RP-HPLC column with detection at 220 nm and eluted with a gradient of Solvent A (H_2_0/0.1% TFA, v/v) and Solvent B (CH_3_CN/0.1% TFA, v/v). The crude peptide (150 mg) was dissolved in a DMF/H_2_O solution (1∶5 v/v, 25 mL), applied to the column equilibrated in Solvent A, and eluted using a linear gradient of (0–70% Solvent B over 1.5 h at a flow-rate of 5 mL/min). Fractions were analyzed for purity using an analytical C_18_ RP-HPLC column employing a linear gradient (0–100% Solvent B over 60 min at a flow-rate of 1 mL/min) and detected at 220 nm. Fractions containing peptide product of at least 90% purity were pooled and concentrated by lyophilization to yield 35 mg (25% yield) of a white solid. A small amount (< 1 mg) of the resulting purified peptide was dissolved in 10 µl of 0.1% TFA/CH_3_CN and diluted 1∶50 in a mixture of CH_3_CN/H_2_O (1∶1 v/v) prior to MS analysis. MS was performed using a 50 µL injection and collecting 3000 scans. ESI-MS: calcd for C_123_H_190_N_32_O_41_ [M+2H]^2+^ 1386.6945, found 1386.6894.

#### 2.4.2 Synthesis of OXY(Gly)_4_-PDAQLVPGINGKAIHLVNNESSE-OH

Starting with 50.0 µmol of the previously described peptide on resin, the free amino terminus was acylated with the 6OXY(Gly)_4_OH analog (31 mg, 50 µmol, 1 eq) catalyzed by DIEA (8.6 µL, 5.0 µmol, 0.1 eq) in DMF (5 mL) for 10 h. After acylation was judged complete by ninhydrin analysis, the resin bound peptide was washed thoroughly with CH_2_Cl_2_ and dried *in vacuo* for 4 h. Peptide cleavage, purification, and analysis was carried out in the same fashion as described above. Synthesis resulted in 33 mg (22% yield) of a white solid. ESI-MS: calcd for C_131_H_202_N_36_O_45_ [M+2H]^2+^ 1500.7480, found 1500.7429.

### 2.5 Serum IgG antibody titers

ELISA plates were coated with 5 ng/well of OVA conjugates, 6OXY-TT, unconjugated OVA or TT in carbonate buffer at pH 9.6 and blocked with 1% gelatin. Primary antibodies were incubated with anti-rat or anti-mouse IgG antibodies (Jackson ImmunoResearch Laboratories, West Grove, PA) to measure oxycodone-specific serum IgG antibody titers as described previously [14] and TT-specific serum IgG antibody titers.

### 2.6 Distribution studies

Serum and brain oxycodone concentrations were measured by gas chromatography coupled to mass spectrometry as previously described [14]. The reported drug concentrations represent the total drug (protein or antibody-bound as well as free) in each sample.

### 2.7 Analysis of hapten-specific B cells

First, all cells from lymph nodes and spleens are incubated with the 6OXY hapten conjugated to the fluorescent protein PE. Then, cells of interest are isolated through anti-PE antibodies coupled to magnetic microbeads and positive selection by magnetic columns as described previously [20]. To distinguish between B cells that bind either 6OXY or PE alone, B cells are pre-incubated with a decoy reagent consisting of PE conjugated to the fluorochrome Alexa Fluor 647 (AF647) prior to incubation with 6OXY-PE. Using a flow cytometer, AF647-PE and 6OXY-PE different emissions allow separating B cells bound to both PE or 6OXY-PE from B cells bound to the 6OXY-PE conjugate alone. Hapten-specific B cells are further characterized through staining with fluorochrome-coupled antibodies specific B cell surface markers as described [20].

#### 2.7.1 Lymph node and spleen tissue isolation

Mesenteric and peripheral lymph nodes and spleens were collected from each mouse, mechanically dissociated, and then enzymatically disaggregated as described previously [20]. Samples were centrifuged at 1600 RPM at +4°C and resuspended to a final volume of 200 µl sorter buffer (DPBS + 2% fetal bovine serum, 0.1% sodium azide) including Fc block (2.4G2, BioXCell, West Lebanon, NH).

#### 2.7.2 Enrichment and positive isolation of 6OXY-specific B cells

Samples were incubated with 5nM Decoy Alexa Fluor 647 conjugated to PE (AF647-PE) at room temperature for 5 min as described [20]. Then samples were incubated with 5 nM 6OXY-PE for 25 min at +4°C. Samples were washed in ice-cold sorter buffer and resuspended to a final volume of 200 µl using sorter buffer, then mixed with 25 µl of anti-PE conjugated magnetic beads (Miltenyi Biotech, Inc, Auburn, CA) and incubated for 15 min at +4°C. The cells were resuspended in sorter buffer and passed through a magnetized LS column (Miltenyi Biotech). For each sample, bound fractions containing B cells bound to PE and 6OXY-PE were centrifuged at 1600 RPM for 5 min at +4°C and resuspended in a final volume of 100 µl of sorter buffer. From each sample, 5 µl were added to 200 µl of lymphocyte fluorescent counting beads at a concentration of 200,000 beads/ml (Accucheck, Invitrogen, Frederick, MD) to calculate numbers of hapten-specific B cells in each sample as described [20].

#### 2.7.3 B cell staining

Cell suspensions were incubated, for 25 minutes on ice, with fluorochrome labeled anti-mouse antibodies for the following B cell surface markers: FITC anti-GL7, PE-Cy7 anti-B220, APC anti-IgM, AF700 anti-CD38, eF450 anti-IgD; and for the following APC-eF780 labeled anti-mouse antibodies for non-B cell surface markers: anti-CD90.2, anti-CD11c, anti-Ly-6G and anti-F4/80. All antibodies were from eBioscience (San Diego, CA) with the exception of FITC anti-GL7 (BD Pharmingen). Cells were fixed in formaldehyde (Cytofix/Cytoperm, BD biosciences, San Diego, CA) for 30 minutes on ice, washed with permeabilization buffer (BD biosciences, San Diego, CA), and incubated with Pacific Orange labeled surface/ intracellular marker anti-mouse anti-Ig heavy and light chain as described [17], [20]. Compensation was prepared for each individual fluorochrome-coupled antibody used in the staining mixture.

#### 2.7.4 Flow cytometry

Flow cytometry studies were performed on a 4-laser (355 nm, 405 nm, 488 nm, 633 nm) LSR II device (BD Biosciences) and data processed with FlowJo software (Tree Star, Ashland, OR).

#### 2.7.5 Gating strategy to analyze 6OXY-specific antibody secreting B cells

First, a singlet gate is applied to remove any cells that have aggregated. Next, total B cells are gated as cells that express immunoglobulin positive (Ig^+^) but not the non-B cell markers CD90.2 (T cells), Gr-1 (neutrophils), CD11.c (dendritic cells) and F4/80 (macrophages). To differentiate between B cells that bind 6OXY from PE-binding B cells, B cells were further distinguished by using AF647-PE, which fluorescences at a wavelength of 670 nm compared to the PE alone at 575 nm. Hapten-specific B cells were further identified as either Ig^high^ antibody secreting cells (ASC) or B220^high^ non-antibody secreting cells (non-ASC) as previously described [18], [20].

### 2.8 Animal studies

#### Ethics statement

All studies were approved by the Minneapolis Medical Foundation and the University of Minnesota Animal Care and Use Committees (protocols #08-10 and #12-04). All animals were euthanized by CO_2_ inhalation using IACUC approved chambers, and efforts were made to minimize suffering and discomfort.

#### 2.8.1 Effect of carrier in mice

Male BALB/c mice (Harlan Laboratories, Madison, WI), age 5–7 weeks at arrival, were group housed with a 12/12 hrs standard light/dark cycle and fed standard mouse chow. Mice were immunized with 25 µg of 6OXY-TT, 6OXY-TT peptide or TT (n =  5-6) on days 0, 14 and 28 administered s.c. in a final volume of 0.2 ml containing alum adjuvant as described [14]. A week after the last immunization, on day 35, the efficacy of vaccination in preventing oxycodone antinociception was tested on a hot plate set at 54°C to determine the % maximal possible effect (MPE %) as described before [14]. Immediately after reaching nociception endpoints, mice were euthanized and brains collected to measure oxycodone concentration, to test the efficacy of vaccination in preventing oxycodone distribution to the brain as described [14], [20]. In all studies involving the hot plate test and measurement of oxycodone brain concentration, experimenters were blind to treatment.

#### 2.8.2 Effect of adjuvant and route in mice

In a separate cohort, male BALB/c mice (n =  5) were immunized with 25 µg of 6OXY-nKLH on days 0, 14 and 28 administered either: s.c. in undiluted alum; s.c. in alum + 2 µg of MPLA; s.c. in 2 µg of MPLA; s.c. in 20 µg of MPLA and i.p. in 2 µg of MPLA. A week after the last immunization, on day 35, mice were euthanized to measure oxycodone-specific serum IgG antibody titers.

#### 2.8.3 Effect of adjuvant in mice

In a separate cohort, male BALB/c mice (n =  4) were immunized with 25 µg of 6OXY-TT on days 0, 14 and 28 administered s.c. using either undiluted alum or 2 µg of MPLA adjuvant. A week after the last immunization, prior to euthanasia by CO_2_, blood was obtained by facial vein sampling and then peripheral lymph nodes and spleens were collected by post-mortem dissection to perform B cell analysis as described previously [20].

#### 2.8.4 Effect of TLR4 signaling and genotype in mice

In a separate study, C57BL/6, C57BL/10ScSnJ and C57BL/10ScNJ Trl4 knockout (TLR4^-/-^) mice (The Jackson Laboratory, Bar Harbor, ME) were immunized with 25 µg of 6OXY-nKLH on days 0, 21 and 42 administered s.c. using undiluted alum adjuvant (n = 6). Serum IgG antibody titers were evaluated at 3 weeks after each vaccination through facial vein blood sampling, respectively at days 21, 42 and 63. At 5 weeks after the last vaccination, on day 77, peripheral lymph nodes and spleens were collected by post-mortem dissection to perform B cell analysis as described before [20].

#### 2.8.5 Effect of adjuvant and route in rats

Male Holtzman rats (Harlan Laboratories, Madison, WI), age 6–7 weeks at arrival, were housed with a 12/12 hrs standard light/dark cycle and fed standard rat chow. Rats (n = 3-5) were immunized with 25 µg of TT or 6OXY-TT on days 0, 21 and 42 administered s.c. in alum adjuvant or i.p. in Freund’s adjuvant as described [14]. A week after the last immunization, the efficacy of vaccination in preventing oxycodone antinociception was tested on a hot plate set at 54°C to determine the % maximal possible effect (MPE %) as described before [14].

#### 2.8.6 Effect of carrier, adjuvant and route in rats

In a separate cohort, male Holtzman rats were immunized with 100 µg of 6OXY-nKLH, 6OXY-dKLH or KLH (n =  7) on days 0, 21 and 42 administered s.c. in alum adjuvant or i.p. in Freund’s. In the Freund’s group, Complete formulation was used for the first injection followed by boosts using Incomplete formulation as described previously [15]. A week after the last immunization, we measured the efficacy of vaccination in preventing oxycodone antinociception, and immediately after reaching nociception endpoints, rats were euthanized to measure oxycodone serum and brain concentration as described before [14].

### 2.9 Statistical analysis

In all studies, unless specified, the mean oxycodone MPE%, oxycodone brain concentration and oxycodone-specific serum antibody titers were compared by one-way ANOVA followed by Bonferroni post hoc test across multiple groups or by Dunnett post hoc test when comparing to unconjugated protein carrier control. When a specific experiment involved only two groups, means were analyzed by unpaired T test. In the TLR4^-/-^ mouse experiment, a two-way (genotype x time) repeated measure ANOVA followed by Bonferroni post hoc test was used to compare mean oxycodone-specific serum antibody titers across mouse strains at 3, 6 and 9 weeks. In B cell studies, the mean numbers of 6OXY-specific B cells were compared by unpaired T test between groups, while the relationship between B cells and serum antibody titers was analyzed by linear regression. All analyses were performed with GraphPad Prism (version 5.0).

## Results

### 3.1 Pre-clinical efficacy of the 6OXY-TT and 6OXY-TT derived peptide immunogens in mice

**Figure 1:**
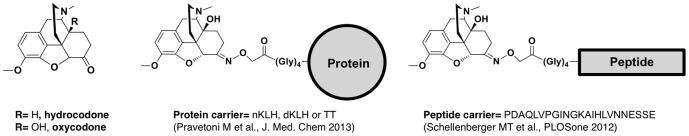
Drug and immunogen structures. Oxycodone and hydrocodone free drugs, and representation of hapten conjugated to carriers. The lead 6OXY hapten was conjugated to native KLH decamer (nKLH), GMP grade KLH dimer (dKLH), tetanus toxoid (TT) and a TT-derived peptide.

**Figure 2:**
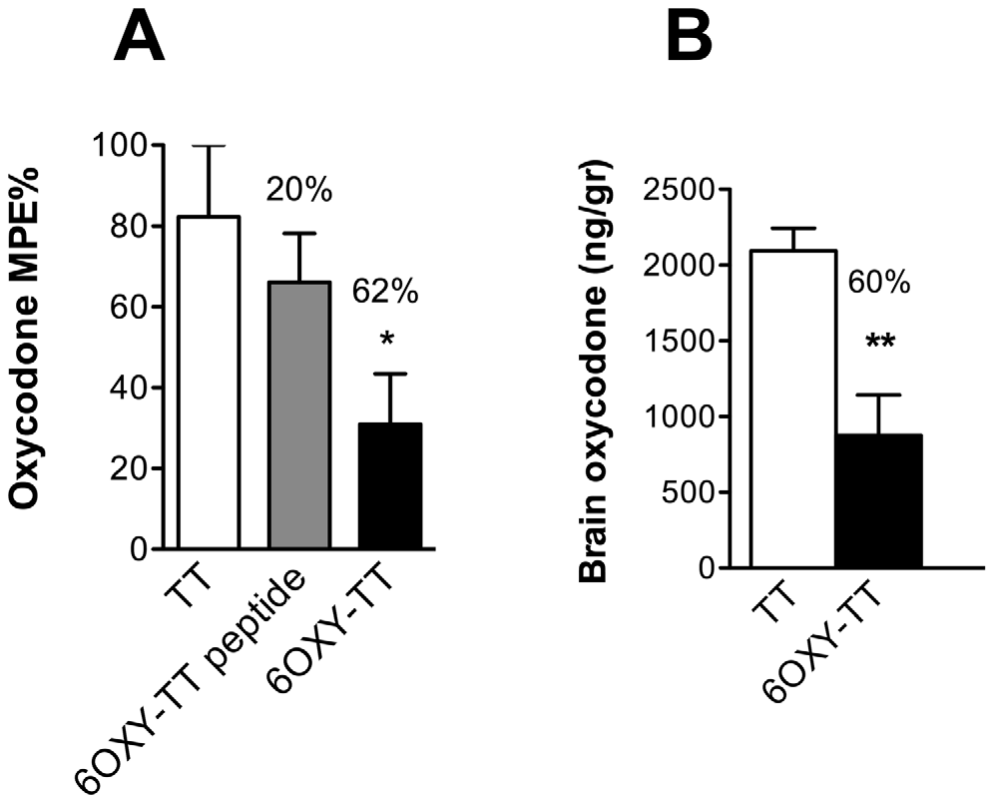
The ability of vaccination with different carrier-containing immunogens in preventing oxycodone nociception and its distribution to the brain in BALB/c mice. A) Oxycodone antinociception in mice immunized with either 6OXY-TT, 6OXY-TT peptide or unconjugated TT. The % maximal possible effect (MPE%) is calculated as (post-drug – baseline)/ (maximal cutoff-baseline) * 100; and B) Oxycodone distribution to the brain in the same mice as in previous panel. In both panels, percent (%) decrease compared to TT control is shown on top of each group. Statistical symbols: * p< 0.05 compared to TT control.

To test if the 6OXY hapten conjugated to the whole TT protein or to a TT-derived peptide (Figure 1) was as effective as the lead 6OXY-nKLH immunogen in preventing oxycodone brain distribution and antinociception, we tested the effect of vaccination on the distribution of a 2.25 mg/kg s.c. dose of oxycodone and its effect on antinociception as previously described [14]. The 6OXY-TT significantly decreased oxycodone antinociception and brain distribution compared to control mice immunized with the unconjugated TT carrier protein (Figure 2). The magnitude of effect in reducing oxycodone distribution and antinociception was comparable to 6OXY-nKLH [14], while the 6OXY-TT derived peptide was not effective in blocking oxycodone antinociception (Figure 2). Indeed, 6OXY-TT elicited oxycodone-specific serum IgG antibody titers of 71,450±12,800 (mean±SEM, n =  5) in contrast to no detectable (<200) titers in mice immunized with 6OXY-TT derived peptide (n =  6).

### 3.2 Pre-clinical efficacy of 6OXY-TT in rats

**Figure 3:**
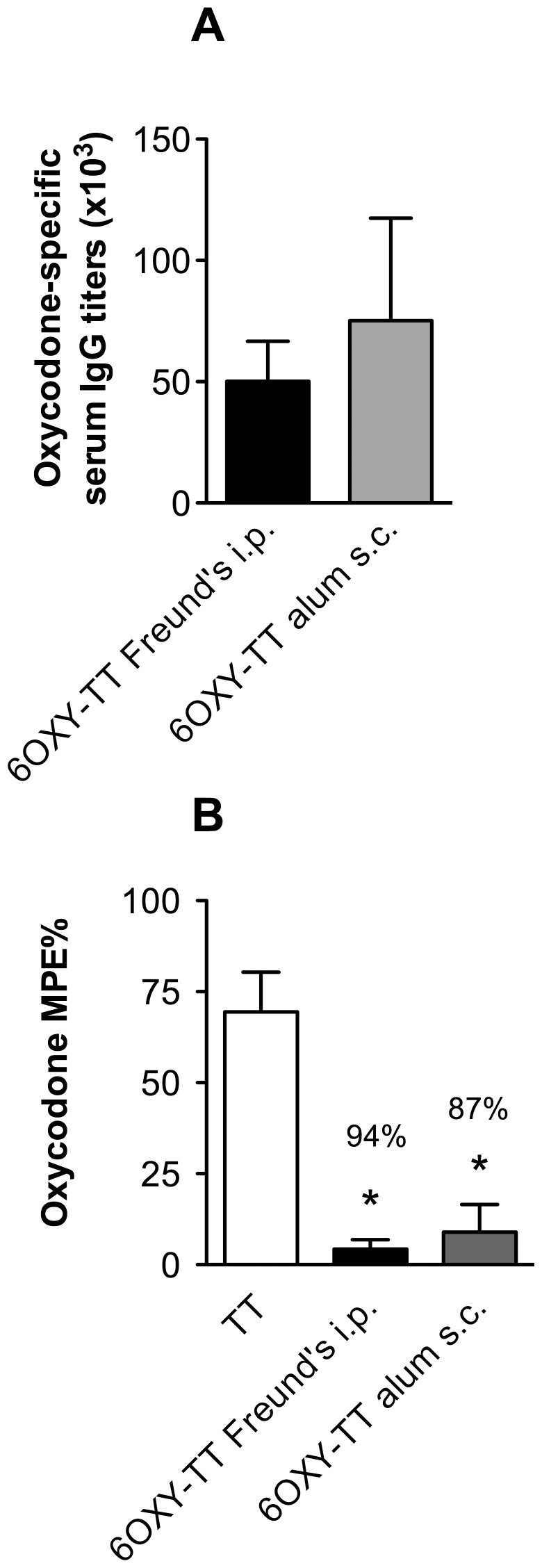
Evaluation of 6OXY-TT in rats. A) oxycodone-specific serum antibody titers in rats immunized with 6OXY-TT using alum s.c. or Freund’s i.p.; and B) their ability in preventing oxycodone-induced antinociception, shown as % decrease compared to TT control group. Statistical symbols: * p< 0.05 compared to TT control.

To confirm that 6OXY-TT administered s.c. absorbed in alum adjuvant was the most promising combination of carrier and adjuvant, we compared this formulation to the standard Freund’s adjuvant using the i.p. route of administration in rats. Immunization with 6OXY-TT was equally effective in generating oxycodone-specific serum IgG antibody titers that prevented oxycodone-induced nociception whether administered s.c. in alum or i.p. in Freund’s adjuvant (Figure 3).

### 3.3 Pre-clinical efficacy of 6OXY-dKLH in rats

**Figure 4:**
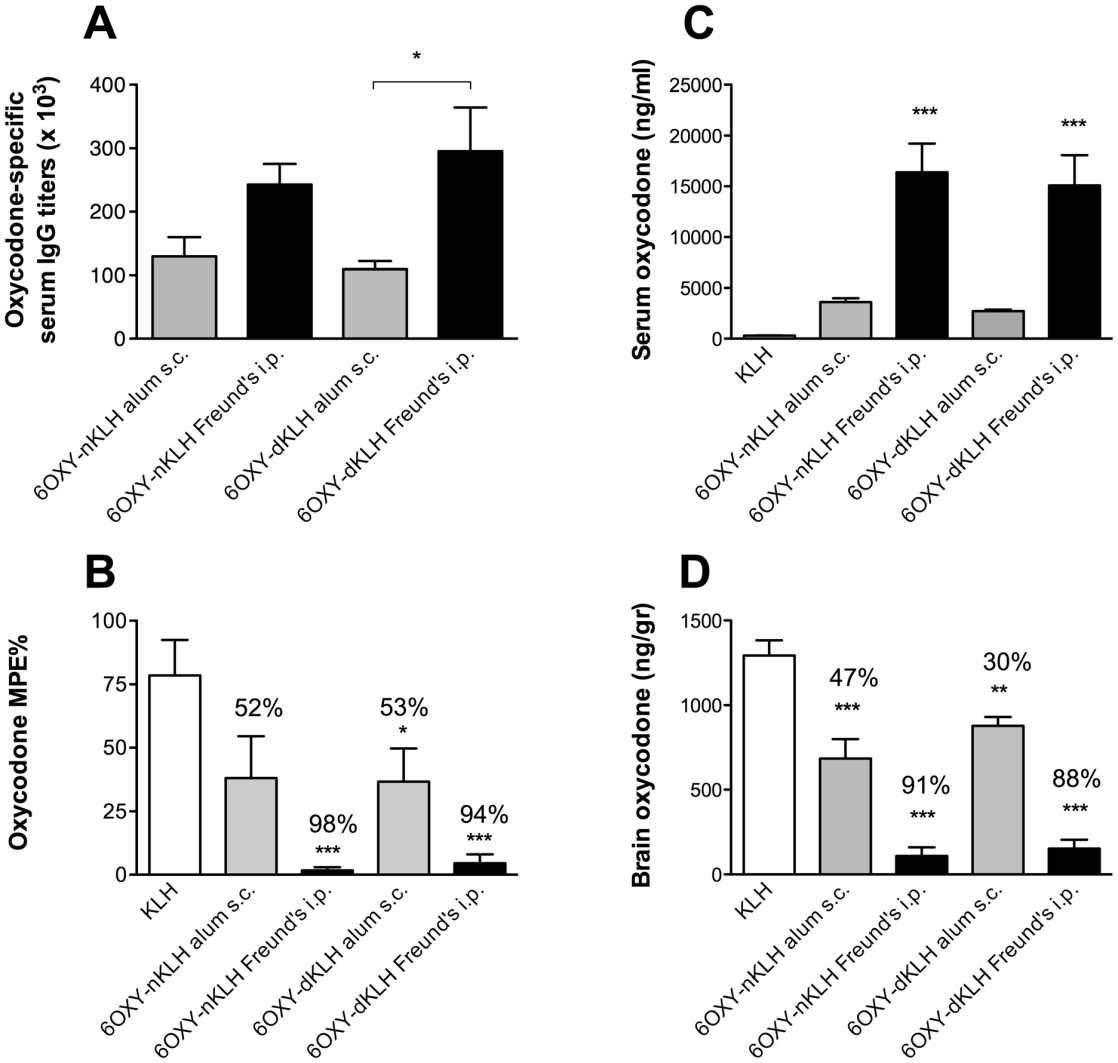
Evaluation of GMP grade dKLH in rats. A) oxycodone-specific serum antibody titers in rats immunized with 6OXY-nKLH or 6OXY-dKLH using alum s.c. or Freund’s i.p.; B) their ability in preventing oxycodone-induced antinociception; C) their effect on oxycodone serum concentration; and D) vaccination effect in preventing brain distribution. Percent (%) decrease compared to nKLH control is shown on top of each group. Statistical symbols: * p< 0.05, **p< 0.01 and *** p< 0.001 compared to KLH control. Brackets to indicate between groups differences.

Next we tested if conjugation of 6OXY to the GMP KLH dimer (∼1,600 kDa) was as effective as to the previously characterized native KLH decamer (∼5–8 million Da). This study was important because it has been shown that different KLH forms or formulation may affect T cell-dependent generation of serum antibody responses [23]. Here, we found that 6OXY-nKLH and 6OXY-dKLH elicited equivalent serum antibody titer responses, blockage of oxycodone behavioral effects and effects on oxycodone distribution if administered using the same route and adjuvant (Figure 4). Instead, using both immunogens, Freund’s i.p. was more effective than alum s.c. in inducing functionally-relevant antibody (Figure 4). We suggest that use of KLH forms should be carefully evaluated along with different adjuvants to optimize serum antibody responses.

### 3.4 Pre-clinical efficacy of MPLA, alone or in combination with alum, on the immunogenicity of 6OXY-nKLH in mice

**Figure 5:**
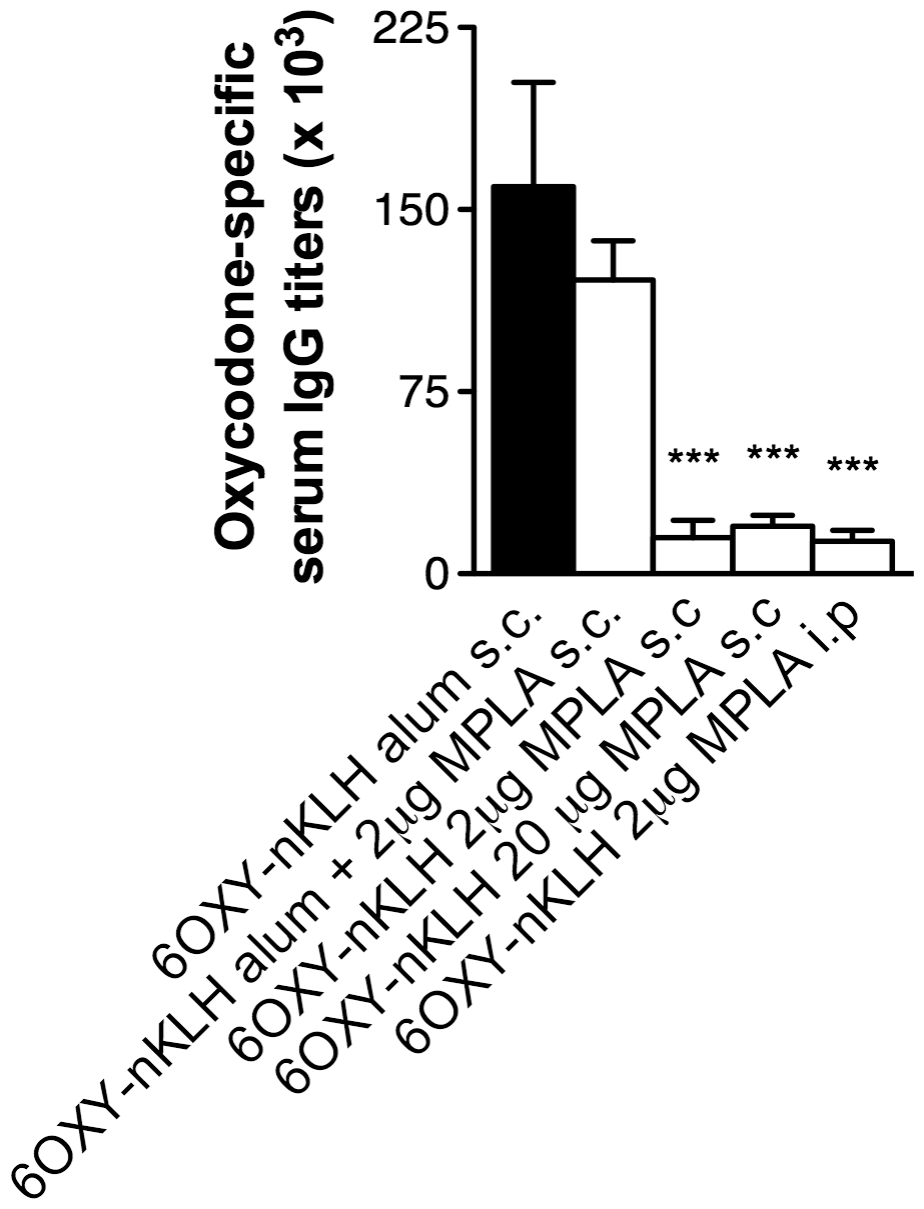
MPLA decreases immunogenicity of 6OXY-nKLH vaccine. Oxycodone-specific serum antibody titers in BALB/c mice immunized with alum adjuvant alone, MPLA alone or in combination with alum. Statistical symbols: *** p< 0.001 compared to 6OXY-nKLH in alum s.c. as control.

Next, we evaluated the effect of the recently approved TLR4 agonist monophosphoryl lipid A (MPLA) adjuvant, alone or in combination with alum, on the immunogenicity of 6OXY-nKLH in mice. We choose mice to provide a cost-effective screening of adjuvant formulations. MPLA elicited lower adjuvant effects on 6OXY-nKLH immunogenicity when administered by the s.c. or i.p routes compared to vaccination using alum s.c. (Figure 5). Combining MPLA with alum showed a slightly lower response, albeit not significant, relative to alum alone (Figure 5).

### 3.5 Pre-clinical efficacy of MPLA on the immunogenicity of 6OXY-TT in mice

**Figure 6:**
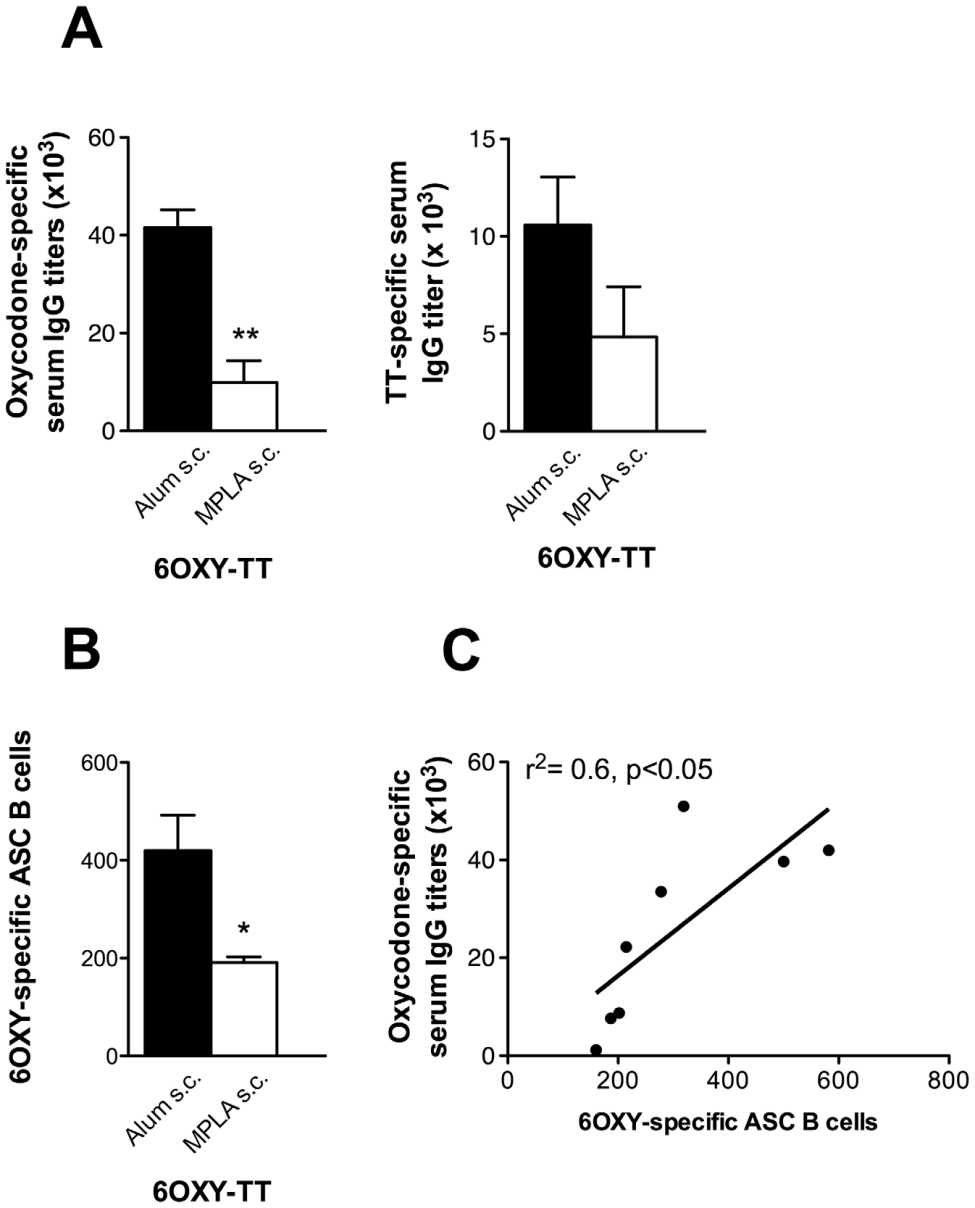
MPLA decreases immunogenicity of 6OXY-TT vaccine. A) Oxycodone-specific and TT-specific serum IgG antibody titers in mice vaccinated s.c. with 6OXY-TT in alum or MPLA adjuvants; B) Number of 6OXY-specific antibody secreting B cells (ASC) in mice immunized s.c. with 6OXY-TT using alum or MPLA; and C) Relationship between oxycodone-specific IgG titers and 6OXY-specific antibody secreting B cells including all subjects immunized with 6OXY-TT. Statistical symbols: * p< 0.05 and **p< 0.01 between groups.

In a separate cohort of mice, we compared the effect of MPLA to alum adjuvant on the immunogenicity of 6OXY-TT to test if MPLA effects were specific to the nKLH carrier or generalized to other carrier proteins. 6OXY-TT elicited higher oxycodone-specific IgG titers using alum adjuvant than when using MPLA (Figure 6, panel A). Similarly, in mice immunized with 6OXY-TT, alum showed a trend of eliciting higher TT-specific serum IgG titers compared to MPLA (Figure 6, panel B). Also, the MPLA adjuvant elicited lower 6OXY-specific Ig^high^ antibody-secreting B cells compared to alum in mice vaccinated with 6OXY-TT (Figure 6, panel B) and the relationship between oxycodone-specific serum IgG antibody titers and 6OXY-specific Ig^high^ B cells was significant (p< 0.05, Figure 6, panel C). This experiment provided proof-of-concept that detecting hapten-specific B cells in immunized mice correlates with other *in vivo* measures of vaccine efficacy and that analysis of B cells may confirm vaccine success and failure and may help rational vaccine design.

### 3.6 Immunogenicity of 6OXY-KLH in TLR4-deficient mice

**Figure 7:**
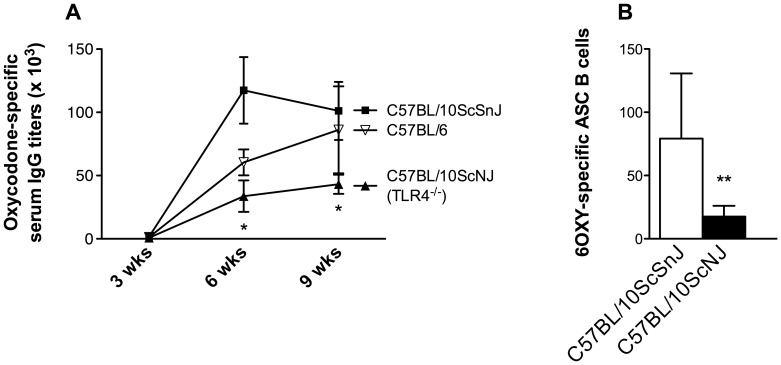
Evaluation of 6OXY-nKLH in different mouse strains. The immunogenicity of 6OXY-nKLH in C57Bl/10ScNJ TRL4^-/-^ mice was reduced versus C57Bl/10ScSnJ wt controls. A) Oxycodone-specific serum IgG antibody titers in mice vaccinated s.c. with 6OXY-nKLH in alum adjuvant; and B) Number of 6OXY-specific antibody secreting B cells in C57Bl/10ScNJ TRL4^-/-^ and C57Bl/10ScSnJ wt mice immunized s.c. with 6OXY-nKLH in alum. Statistical symbols: * p< 0.05 and **p< 0.01 compared to C57Bl/10ScSnJ wt control mice.

In the previous experiment the TLR4 agonist MPLA induced lower oxycodone-specific serum antibody responses to 6OXY-nKLH and 6OXY-TT compared to alum adjuvant. These data suggest that MPLA activation of Th_1_ responses, or alternatively TLR4 signaling, may negatively affect the immunogenicity of oxycodone conjugate vaccines. To test this alternative hypothesis we evaluated the immunogenicity of 6OXY-nKLH in alum in C57Bl/10ScNJ TRL4^-/-^ mice versus C57Bl/6J and C57Bl/10ScSnJ wild-type (wt) controls. Two-way (genotype x time) repeated measures ANOVA showed a significant effect of genotype (F_(2,32)_ = 4.21; p< 0.05) and time (F_(2,32)_ = 15.41; p< 0.001) on oxycodone-specific serum antibody titers. At 6 and 9 weeks after the first immunization, on day 42 and 63, TLR4^-/-^ mice showed lower oxycodone-specific titers than their C57Bl/10ScSnJ wt controls (Figure 7, panel A). Five weeks after the last immunization, on day 77, TLR4^-/-^ mice showed significantly less 6OXY-specific Ig^+^ antibody-secreting B cells than C57Bl/10ScSnJ wt controls (Figure 7, panel B).

## Discussion

This study explored the pre-clinical efficacy of an oxycodone vaccine using clinically approved carriers and adjuvants to provide a formulation suitable for human use. Testing the role of hapten, carrier and adjuvant is important to achieve optimal generation of drug-specific serum antibodies. Recent high-throughput studies of nicotine vaccines showed how critical it is to screen for both quantity and quality of the immune response against drugs of abuse to select a vaccine candidate [5], [9].

In our current study, we found that 6OXY-TT and 6OXY-dKLH were as effective as the previously characterized 6OXY-nKLH, while the 6OXY hapten conjugated to a TT-derived peptide was not effective in preventing oxycodone-induced antinociception. Use of 6OXY-TT s.c. absorbed on alum adjuvant provided similar protection to 6OXY-TT administered i.p. with Freund’s adjuvant in rats. The TLR4 agonist MPLA induced lower oxycodone-specific serum antibodies in mice vaccinated with 6OXY-nKLH relative to alum adjuvant. Similarly, in mice immunized s.c. with 6OXY-TT in MPLA, oxycodone- and TT-specific serum antibodies were diminished compared to 6OXY-TT in alum. MPLA also induced lower numbers of 6OXY-specific antibody-secreting B cells in mice immunized with 6OXY-TT relatively to alum. The TLR4 agonist MPLA adjuvant, known to induce strong Th_1_ responses, was not as effective as alum suggesting that responses to 6OXY-nKLH and 6OXY-TT may depend on Th_2_ activation. Also, the immunogenicity of 6OXY-nKLH was decreased in TLR4-deficient mice, suggesting that responses to 6OXY-nKLH and 6OXY-TT may require intact TLR4 signaling. These data provided evidence, and potential underlying mechanisms, of how clinically approved carriers and adjuvants mediate the pre-clinical efficacy of an oxycodone vaccine.

Vaccination with the lead 6OXY hapten conjugated to TT elicited equivalent responses to the previously characterized immunogen containing nKLH, and prevented oxycodone distribution to brain and its antinociceptive effects in mice and rats. TT has been successfully used as a carrier for heroin conjugate vaccines [24], [25] and this study extended its use to vaccines against prescription opioids. Similarly, an immunogen containing the GMP grade dimer KLH provided equivalent immunogenicity and efficacy as the previously characterized immunogen containing the multi-subunit native KLH. Selection of appropriate carrier is critical to facilitate translation of lead immunogens and to match GMP requirements. KLH-containing immunogens generated higher titers than TT-containing immunogens in both mice and rats. This apparent discrepancy may simply reflect ELISA detection using OVA-containing immunogens. In fact, functional evaluation of these immunogens showed that nKLH, dKLH and TT provided equivalent efficacy against oxycodone distribution to brain or its behavioral effects, suggesting that the 6OXY hapten is well suited for flexible immunogen designs and it can be attached to different carrier proteins.

KLH and TT have been extensively characterized in animal and human studies or clinical practice. KLH has been successfully used as a carrier in clinical trials of conjugate vaccines against carbohydrates expressed on the surface of metastatic breast and prostate cancers [16], [26]–[29]. TT is commonly used in various conjugate vaccines or multivalent immunization strategies such as vaccination against diphtheria, tetanus, and acellular pertussis (DPaT). TT may be a valuable carrier for drug haptens since it has been shown that previous exposure to TT did not interfere with hapten-specific serum antibody responses to a subsequent immunization with a benzopyrene-based hapten conjugated to TT [21]. Other carrier proteins have been safely used in clinical evaluation of addiction vaccines including recombinant cholera toxin B subunit [30], [31], *Pseudomonas Aeruginosa* recombinant exoprotein A [32], [33], and virus-like particles [34], [35].

In contrast to whole proteins, a TT-derived peptide did not provide an effective carrier for the oxycodone-based hapten. This finding was surprising because the same TT-derived peptide was shown to be an effective carrier for a benzopyrene hapten [21]. Peptides have been previously shown to be suitable carriers for nicotine haptens [36]. These data suggest that peptide carriers should be evaluated for each hapten, since their efficacy may vary greatly according to host strain and genetic background. Peptide carriers may be more effective when combined with functionalized nanocarriers, liposomes or lipid-based adjuvants [25]. Specifically, synthetic peptides may provide GMP grade components for more complex delivery platforms.

The recently clinically approved MPLA adjuvant did not elicit higher serum IgG antibody titers than alum. Using either the 6OXY-nKLH or the 6OXY-TT immunogens, we found that MPLA alone or in combination with alum was negatively associated with oxycodone-specific serum antibody titers. This finding is in contrast to a recent study, where MPLA improved the immunogenicity of a TT-containing heroin vaccine [25]. The effect of adjuvants on the immunogenicity of each conjugate vaccine may differ for various reasons. Adjuvants may have species- and strain-dependent efficacy and also different effects on vaccines containing haptens of different design. For instance, different adjuvants may induce activation of Th_1_ or Th_2_ responses, which may provide vaccine-specific effects on generation of optimal serum antibody levels against drugs of abuse. In this study, MPLA, known to induce Th_1_ activation, was no more effective than alum, a Th_2_ adjuvant, in inducing serum antibody responses to 6OXY-nKLH and 6OXY-TT. Also, in mice immunized with 6OXY-TT, MPLA was less effective in inducing hapten-specific antibody secreting B cells compared to alum adjuvant. Similarly, the Th_1_ adjuvant CpG mixed with alum was not as effective as alum alone at eliciting serum antibody titers in mice vaccinated with a heroin conjugate vaccine [37]. In contrast, the CpG adjuvant added to alum increased the immunogenicity of a nicotine conjugate vaccine compared to alum alone in mice and non-human primates [9]. Multiple adjuvant formulations are available and it is often difficult to compare efficacy across studies or species. In our study, the 6OXY-nKLH, 6OXY-dKLH and 6OXY-TT administered in alum adjuvant, were equally effective in Holtzman rats, and BALB/c, C57Bl/6 and C57Bl/10ScSnJ mice. We acknowledge that pre-clinical efficacy in mice and rats may not necessarily reflect vaccine efficacy in humans. However, these data suggested that alum is an effective and safe first choice adjuvant to test oxycodone conjugate vaccines in humans because results are consistent across species and strains.

The generation of drug-specific serum antibody is the result of T cell-dependent B cell activation, which may be affected by hapten design, carrier and adjuvant. Recently, we have shown that an increased number of hapten-specific naïve and early activated B cells, before and shortly after immunization with oxycodone conjugate vaccines, is associated with increased immunogenicity and vaccine efficacy against oxycodone [20]. In the current study, in mice vaccinated with 6OXY-TT, the oxycodone-specific serum IgG antibody titers correlated with the numbers of 6OXY-specific antibody-secreting B cells. The significant relationship between oxycodone-specific serum IgG antibody titers and the number of 6OXY-specific antibody secreting B cells suggested that the number of hapten-specific activated B cells may be used to study the individual variability observed in immunized subjects, and may be a useful endpoint for optimization of vaccine design.

To further evaluate the role of B cell activation, and to gain mechanistic insight in the observed low efficacy of the TLR4 agonist MPLA adjuvant, we investigated the immunogenicity of 6OXY-nKLH in TLR4-deficient mice. Immunization of TLR4-deficient mice with an oxycodone vaccine elicited significantly lower serum antibody titers and antibody secreting B cells compared to wild-type control mice. This preliminary observation suggested that an intact TLR4 signaling pathway may be required for T cell-dependent B cell activation in response to vaccination with 6OXY-nKLH and 6OXY-TT. Recent evidence shows that TLR4 binds opioids [38]. A possible interpretation of our findings is that TLR4 may also modulate B cell responses to drug of abuse haptens, which are structurally-similar to the target drug. Based upon this hypothesis, MPLA binding to TLR4 may affect, or interfere with, recognition of 6OXY-nKLH. An alternative explanation is that TLR4 mediates responses to nKLH or TT that may contain, or act like, endotoxins. As a cautionary note in regards to interpretation of studies involving constitutional knockout mice, the effect of gene deletion on phenotypes may be affected by compensatory mechanisms or by the genetic background of the parent strain [39].

It has been shown that TLR4 gene polymorphisms or TLR4 expression on B cells predict immune responses to TLR4 agonists and vaccines in mice and humans [40]–[44]. Currently, there are no pre-vaccination markers predictive of efficacy of vaccines against drugs of abuse. Based upon our findings, we suggest that subjects with certain mutations in the TLR4, or expressing lower levels of TLR4 on B cells might be less likely to respond to an oxycodone conjugate vaccine.

## Conclusions

Overall, our findings and available literature suggest that vaccines should be carefully evaluated using suitable carriers in multiple adjuvant formulations to provide reliable efficacy. Such studies would provide important information on individualized immunotherapy targeting specific patient populations. Our data showed that analysis of hapten-specific B cells may be used to investigate the mechanisms underlying antibody response to drug addiction vaccines, and this approach may be useful to study the efficacy of different carrier proteins, adjuvant or to help determine the most successful immunization strategy.

## References

[1] UNODC (2012) World Drug Report 2012.

[2] National Survey on Drug Use and Health: National Findings (2013)

[3] Binswanger IA, Blatchford PJ, Mueller SR, Stern MF (2013) Mortality after prison release: opioid overdose and other causes of death, risk factors, and time trends from 1999 to 2009. Ann Intern Med 159: 592–600. 24189594 10.7326/0003-4819-159-9-201311050-00005 PMC5242316

[4] Shen XY, Orson FM, Kosten TR (2012) Vaccines against drug abuse. Clin Pharmacol Ther 91: 60–70. 22130115 10.1038/clpt.2011.281 PMC3345810

[5] Pryde DC, Jones LH, Gervais DP, Stead DR, Blakemore DC, et al (2013) Selection of a novel anti-nicotine vaccine: influence of antigen design on antibody function in mice. PLoS One 8: e76557. 24098532 10.1371/journal.pone.0076557 PMC3788104

[6] Cai X, Whitfield T, Moreno AY, Grant Y, Hixon MS, et al.. (2013) Probing the Effects of Hapten Stability on Cocaine Vaccine Immunogenicity. Mol Pharm. 10.1021/mp400214w PMC3946501 23927436

[7] de Villiers SH, Cornish KE, Troska AJ, Pravetoni M, Pentel PR (2013) Increased efficacy of a trivalent nicotine vaccine compared to a dose-matched monovalent vaccine when formulated with alum. Vaccine 10.1016/j.vaccine.2013.10.051 PMC4019346 24176492

[8] Hu Y, Zheng H, Huang W, Zhang C (2013) A novel and efficient nicotine vaccine using nano-lipoplex as a delivery vehicle. Hum Vaccin Immunother 10. 10.4161/hv.26635 PMC4181017 24091786

[9] McCluskie MJ, Pryde DC, Gervais DP, Stead DR, Zhang N, et al (2013) Enhancing immunogenicity of a 3'aminomethylnicotine-DT-conjugate anti-nicotine vaccine with CpG adjuvant in mice and non-human primates. Int Immunopharmacol 16: 50–56. 23562759 10.1016/j.intimp.2013.03.021

[10] Lockner JW, Ho SO, McCague KC, Chiang SM, Do TQ, et al (2013) Enhancing nicotine vaccine immunogenicity with liposomes. Bioorg Med Chem Lett 23: 975–978. 23313243 10.1016/j.bmcl.2012.12.048 PMC3557556

[11] Chen X, Pravetoni M, Bhayana B, Pentel PR, Wu MX (2012) High immunogenicity of nicotine vaccines obtained by intradermal delivery with safe adjuvants. Vaccine 31: 159–164. 23123021 10.1016/j.vaccine.2012.10.069 PMC3557791

[12] Matyas GR, Mayorov AV, Rice KC, Jacobson AE, Cheng K, et al (2013) Liposomes containing monophosphoryl lipid A: a potent adjuvant system for inducing antibodies to heroin hapten analogs. Vaccine 31: 2804–2810. 23624097 10.1016/j.vaccine.2013.04.027 PMC4120113

[13] Pravetoni M, Keyler DE, Pidaparthi RR, Carroll FI, Runyon SP, et al (2012) Structurally distinct nicotine immunogens elicit antibodies with non-overlapping specificities. Biochem Pharmacol 83: 543–550. 22100986 10.1016/j.bcp.2011.11.004 PMC3259188

[14] Pravetoni M, Le Naour M, Tucker AM, Harmon TM, Hawley TM, et al (2013) Reduced antinociception of opioids in rats and mice by vaccination with immunogens containing oxycodone and hydrocodone haptens. J Med Chem 56: 915–923. 23249238 10.1021/jm3013745 PMC3791856

[15] Pravetoni M, Le Naour M, Harmon T, Tucker A, Portoghese PS, et al (2012) An oxycodone conjugate vaccine elicits oxycodone-specific antibodies that reduce oxycodone distribution to brain and hot-plate analgesia. J Pharmacol Exp Ther 10.1124/jpet.111.189506 PMC3310692 22262924

[16] Musselli C, Livingston PO, Ragupathi G (2001) Keyhole limpet hemocyanin conjugate vaccines against cancer: the Memorial Sloan Kettering experience. J Cancer Res Clin Oncol 127 Suppl 2: R20–26. 11768620 10.1007/BF01470995 PMC12164596

[17] Pape KA, Taylor JJ, Maul RW, Gearhart PJ, Jenkins MK (2011) Different B cell populations mediate early and late memory during an endogenous immune response. Science 331: 1203–1207. 21310965 10.1126/science.1201730 PMC3993090

[18] Taylor JJ, Martinez RJ, Titcombe PJ, Barsness LO, Thomas SR, et al (2012) Deletion and anergy of polyclonal B cells specific for ubiquitous membrane-bound self-antigen. J Exp Med 209: 2065–2077. 23071255 10.1084/jem.20112272 PMC3478923

[19] Taylor JJ, Pape KA, Jenkins MK (2012) A germinal center-independent pathway generates unswitched memory B cells early in the primary response. J Exp Med 209: 597–606. 22370719 10.1084/jem.20111696 PMC3302224

[20] Taylor JJ, Laudenbach M, Tucker AM, Jenkins MK, Pravetoni M (2014) Hapten-specific naive B cells are biomarkers of vaccine efficacy against drugs of abuse. J Immunol Methods 10.1016/j.jim.2014.01.010 PMC4018303 24462800

[21] Schellenberger MT, Grova N, Farinelle S, Willieme S, Revets D, et al (2012) Immunogenicity of a promiscuous T cell epitope peptide based conjugate vaccine against benzo[a]pyrene: redirecting antibodies to the hapten. PLoS One 7: e38329. 22666501 10.1371/journal.pone.0038329 PMC3364213

[22] O'Neill LA, Bryant CE, Doyle SL (2009) Therapeutic targeting of Toll-like receptors for infectious and inflammatory diseases and cancer. Pharmacol Rev 61: 177–197. 19474110 10.1124/pr.109.001073 PMC2846156

[23] Lebrec H, Hock MB, Sundsmo JS, Mytych DT, Chow H, et al (2013) T-cell-dependent antibody responses in the rat: Forms and sources of keyhole limpet hemocyanin matter. J Immunotoxicol 10.3109/1547691X.2013.822948 23961896

[24] Anton B, Leff P (2006) A novel bivalent morphine/heroin vaccine that prevents relapse to heroin addiction in rodents. Vaccine 24: 3232–3240. 16494974 10.1016/j.vaccine.2006.01.047

[25] Matyas GR, Mayorov AV, Rice KC, Jacobson AE, Cheng K, et al (2013) Liposomes containing monophosphoryl lipid A: A potent adjuvant system for inducing antibodies to heroin hapten analogs. Vaccine 10.1016/j.vaccine.2013.04.027 PMC4120113 23624097

[26] Astronomo RD, Burton DR (2010) Carbohydrate vaccines: developing sweet solutions to sticky situations? Nat Rev Drug Discov 9: 308–324. 20357803 10.1038/nrd3012 PMC3878310

[27] Livingston PO, Ragupathi G (2006) Cancer vaccines targeting carbohydrate antigens. Hum Vaccin 2: 137–143. 17012906 10.4161/hv.2941

[28] Miles D, Roche H, Martin M, Perren TJ, Cameron DA, et al (2011) Phase III multicenter clinical trial of the sialyl-TN (STn)-keyhole limpet hemocyanin (KLH) vaccine for metastatic breast cancer. Oncologist 16: 1092–1100. 21572124 10.1634/theoncologist.2010-0307 PMC3228158

[29] Gilewski TA, Ragupathi G, Dickler M, Powell S, Bhuta S, et al (2007) Immunization of high-risk breast cancer patients with clustered sTn-KLH conjugate plus the immunologic adjuvant QS-21. Clin Cancer Res 13: 2977–2985. 17504999 10.1158/1078-0432.CCR-06-2189

[30] Martell BA, Orson FM, Poling J, Mitchell E, Rossen RD, et al (2009) Cocaine vaccine for the treatment of cocaine dependence in methadone-maintained patients: a randomized, double-blind, placebo-controlled efficacy trial. Arch Gen Psychiatry 66: 1116–1123. 19805702 10.1001/archgenpsychiatry.2009.128 PMC2878137

[31] Kosten TR, Rosen M, Bond J, Settles M, Roberts JS, et al (2002) Human therapeutic cocaine vaccine: safety and immunogenicity. Vaccine 20: 1196–1204. 11803082 10.1016/s0264-410x(01)00425-x

[32] Hatsukami DK, Jorenby DE, Gonzales D, Rigotti NA, Glover ED, et al (2011) Immunogenicity and smoking-cessation outcomes for a novel nicotine immunotherapeutic. Clin Pharmacol Ther 89: 392–399. 21270788 10.1038/clpt.2010.317 PMC4106715

[33] Hatsukami DK, Rennard S, Jorenby D, Fiore M, Koopmeiners J, et al (2005) Safety and immunogenicity of a nicotine conjugate vaccine in current smokers. Clin Pharmacol Ther 78: 456–467. 16321612 10.1016/j.clpt.2005.08.007

[34] Cornuz J, Zwahlen S, Jungi WF, Osterwalder J, Klingler K, et al (2008) A vaccine against nicotine for smoking cessation: a randomized controlled trial. PLoS One 3: e2547. 18575629 10.1371/journal.pone.0002547 PMC2432028

[35] Maurer P, Jennings GT, Willers J, Rohner F, Lindman Y, et al (2005) A therapeutic vaccine for nicotine dependence: preclinical efficacy, and Phase I safety and immunogenicity. Eur J Immunol 35: 2031–2040. 15971275 10.1002/eji.200526285

[36] Sanderson SD, Cheruku SR, Padmanilayam MP, Vennerstrom JL, Thiele GM, et al (2003) Immunization to nicotine with a peptide-based vaccine composed of a conformationally biased agonist of C5a as a molecular adjuvant. Int Immunopharmacol 3: 137–146. 12538044 10.1016/s1567-5769(02)00260-6

[37] Bremer PT, Janda KD (2012) Investigating the effects of a hydrolytically stable hapten and a Th1 adjuvant on heroin vaccine performance. J Med Chem 55: 10776–10780. 23134263 10.1021/jm301262z PMC3527012

[38] Wang X, Loram LC, Ramos K, de Jesus AJ, Thomas J, et al (2012) Morphine activates neuroinflammation in a manner parallel to endotoxin. Proc Natl Acad Sci U S A 109: 6325–6330. 22474354 10.1073/pnas.1200130109 PMC3341002

[39] Picciotto MR, Wickman K (1998) Using knockout and transgenic mice to study neurophysiology and behavior. Physiol Rev 78: 1131–1163. 9790572 10.1152/physrev.1998.78.4.1131

[40] Grondahl-Yli-Hannuksela K, Vuononvirta J, Barkoff AM, Viander M, Van Der Meeren O, et al (2012) Gene polymorphism in toll-like receptor 4: effect on antibody production and persistence after acellular pertussis vaccination during adolescence. J Infect Dis 205: 1214–1219. 22383676 10.1093/infdis/jis182

[41] Kimman TG, Banus S, Reijmerink N, Reimerink J, Stelma FF, et al (2008) Association of interacting genes in the toll-like receptor signaling pathway and the antibody response to pertussis vaccination. PLoS One 3: e3665. 18987746 10.1371/journal.pone.0003665 PMC2573957

[42] Schroder NW, Schumann RR (2005) Single nucleotide polymorphisms of Toll-like receptors and susceptibility to infectious disease. Lancet Infect Dis 5: 156–164. 15766650 10.1016/S1473-3099(05)01308-3

[43] Tsukamoto H, Fukudome K, Takao S, Tsuneyoshi N, Ohta S, et al (2013) Reduced surface expression of TLR4 by a V254I point mutation accounts for the low lipopolysaccharide responder phenotype of BALB/c B cells. J Immunol 190: 195–204. 23203928 10.4049/jimmunol.1201047

[44] Yamakawa N, Ohto U, Akashi-Takamura S, Takahashi K, Saitoh S, et al (2013) Human TLR4 polymorphism D299G/T399I alters TLR4/MD-2 conformation and response to a weak ligand monophosphoryl lipid A. Int Immunol 25: 45–52. 22962435 10.1093/intimm/dxs084

